# Apalutamide-Induced Drug Reaction With Eosinophilia and Systemic Symptoms (DRESS) Syndrome in a Patient With Metastatic Prostate Cancer: A Case Report

**DOI:** 10.7759/cureus.117816

**Published:** 2026-10-12

**Authors:** Shawly Kabir, Lynne Cormode

**Affiliations:** 1 Department of Oncology, Ninewells Hospital, Dundee, GBR

**Keywords:** apalutamide, cutaneous adverse drug reaction, dress syndrome, metastatic hormone-sensitive prostate cancer, metastatic prostate cancer

## Abstract

Metastatic prostate cancer is a leading cause of cancer-related morbidity and mortality. Androgen deprivation therapy (ADT) remains the foundation of systemic treatment, with novel androgen receptor inhibitors such as apalutamide increasingly used in patients with metastatic hormone-sensitive prostate cancer (mHSPC). Apalutamide is generally well tolerated. Drug reaction with eosinophilia and systemic symptoms (DRESS) syndrome is not recognized as an established side effect of apalutamide.

We report the case of a man in his seventies who was initiated on apalutamide for mHSPC. Ten days after initiation, he developed a widespread maculopapular rash accompanied by biochemical abnormalities. Clinical assessment suggested a diagnosis of DRESS syndrome, most consistent with the criteria of Bocquet et al. The patient was managed with immediate discontinuation of apalutamide and initiation of high-dose systemic corticosteroids. He improved clinically and biochemically and subsequently transitioned to enzalutamide.

This case highlights the importance of recognizing rare but potentially life-threatening adverse reactions associated with apalutamide.

## Introduction

Metastatic prostate cancer represents one of the leading causes of cancer-related morbidity and mortality worldwide. Androgen deprivation therapy (ADT) remains the cornerstone of systemic treatment and is frequently initiated either with luteinizing hormone-releasing hormone (LHRH) agonists (leuprorelin, goserelin) or antagonists (degarelix, relugolix). The choice between these agents often depends on the cardiovascular risk profile and the potential for tumor flare [1,2].

Apalutamide is a next-generation nonsteroidal androgen receptor inhibitor used in patients with newly diagnosed advanced prostate cancer who are considered hormone-sensitive. By competitively binding to the ligand-binding domain of the androgen receptor, apalutamide prevents androgen-mediated transcriptional activity, thereby reducing tumor cell proliferation and promoting apoptosis [3,4].

Up to 25% of patients can develop cutaneous adverse events, most frequently in the form of maculopapular rashes, typically within the first three months of treatment. The rash is reported to recur in half of patients when rechallenged. These are usually manageable with topical corticosteroids and oral antihistamines, although dose modification or discontinuation is sometimes required [4].

Drug reaction with eosinophilia and systemic symptoms (DRESS) syndrome is a rare but potentially life-threatening drug-induced hypersensitivity reaction. It is characterized by fever, widespread skin eruption, lymphadenopathy, hematological abnormalities such as eosinophilia or atypical lymphocytosis, and internal organ involvement [5].

Emerging reports suggest that apalutamide may be a possible trigger for DRESS syndrome. Here, we describe a case of apalutamide-induced DRESS syndrome diagnosed and managed in Scotland.

## Case presentation

A man in his seventies presented to the emergency department following a syncopal episode. His past medical history was notable for right inguinal hernia repair and obstructive sleep apnea. On admission, he was found to be in urinary retention, which was considered the likely precipitant of his syncopal episode. He also reported several months of left testicular swelling with intermittent discomfort and episodes of hematuria.

Initial investigations revealed a prostate-specific antigen (PSA) level of 490.5 mcg/L (reference range 0-4.9 mcg/L). Ultrasound of the testes demonstrated bilateral hydroceles. Staging studies, including bone scintigraphy (Figure 1) and computed tomography (CT) (Figures 2, 3), confirmed widespread lymphadenopathy and extensive bone and lung metastatic disease, with at least T3 local disease diagnosed at subsequent urology review. The patient declined prostate biopsy but agreed to medical management and was commenced on degarelix. He was referred to oncology for consideration of additional systemic anticancer therapy (SACT).

**Figure 1:**
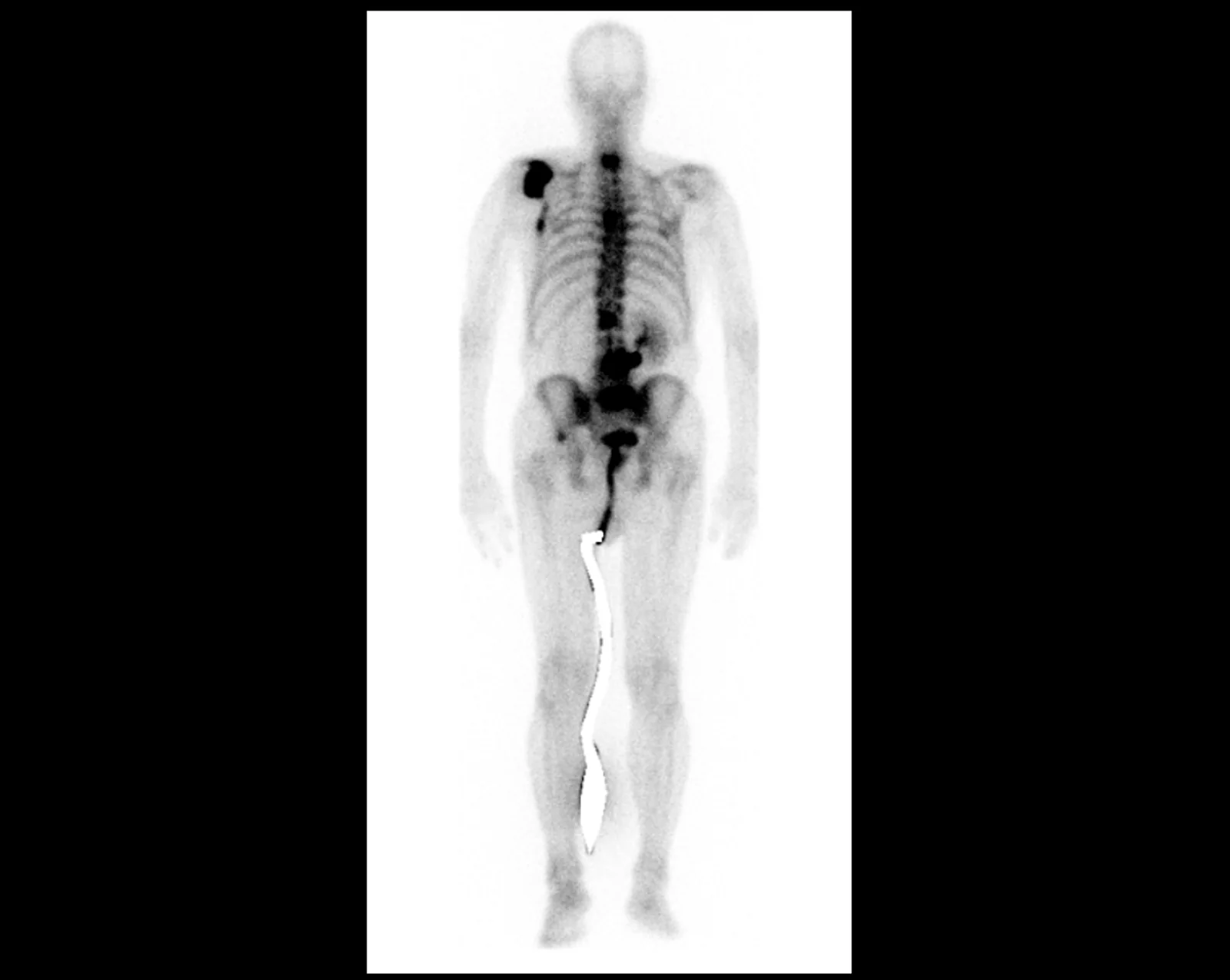
Whole-body nuclear medicine bone scan showing intense tracer accumulation involving the left scapula and left third rib, along with several thoracic and lumbar vertebrae

**Figure 2:**
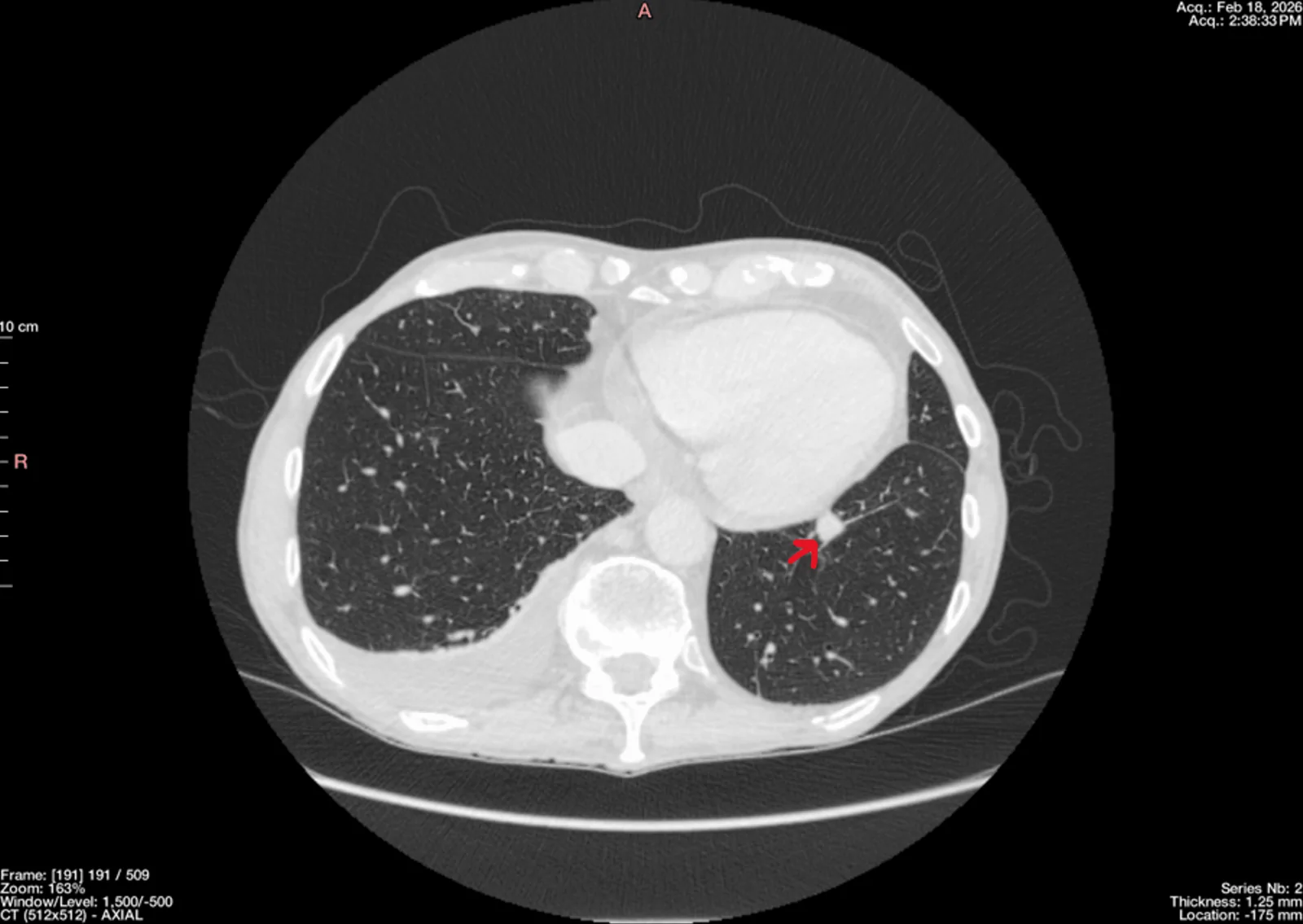
Suspicious metastatic lung nodule (red arrow)

**Figure 3:**
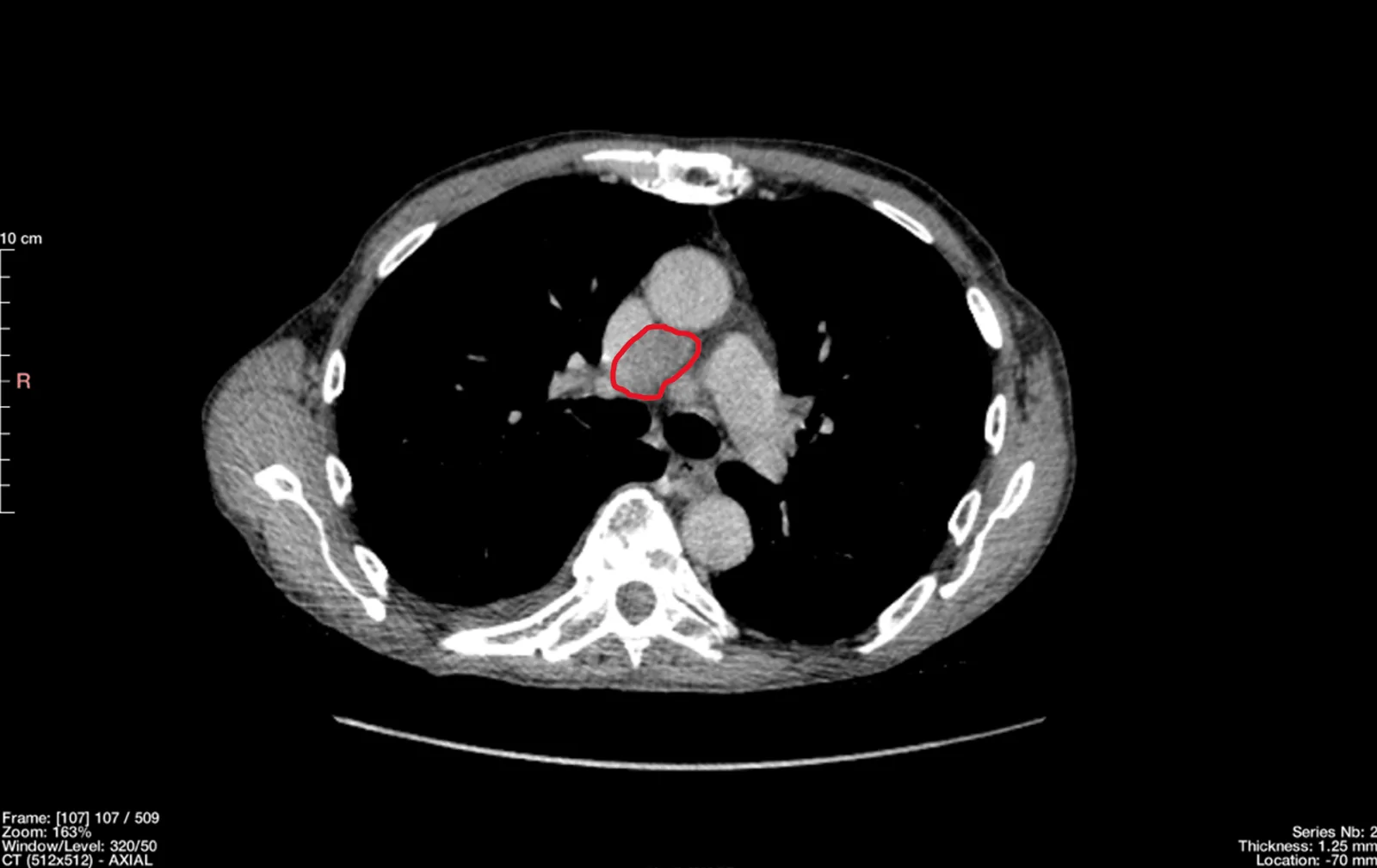
Multiple metastatic mediastinal lymph nodes (circled in red)

Given the hormone-sensitive status of his disease, the patient was initiated on apalutamide (240 mg once daily). Clinical trials have shown that the use of apalutamide with degarelix can improve overall survival in patients with mHSPC [6]. Ten days after starting treatment, he presented to the Oncology Triage Service with a widespread maculopapular rash affecting the chest, abdomen, and legs. Apalutamide was discontinued immediately, and treatment with topical corticosteroids and oral antihistamines was commenced. Additional prescriptions for Dermovate 0.05%, Epimax cream, and QV wash were provided.

However, the rash worsened (Figures 4, 5). The patient reported minimal pruritus and denied mucosal involvement. His medical history revealed no prior drug allergies.

**Figure 4:**
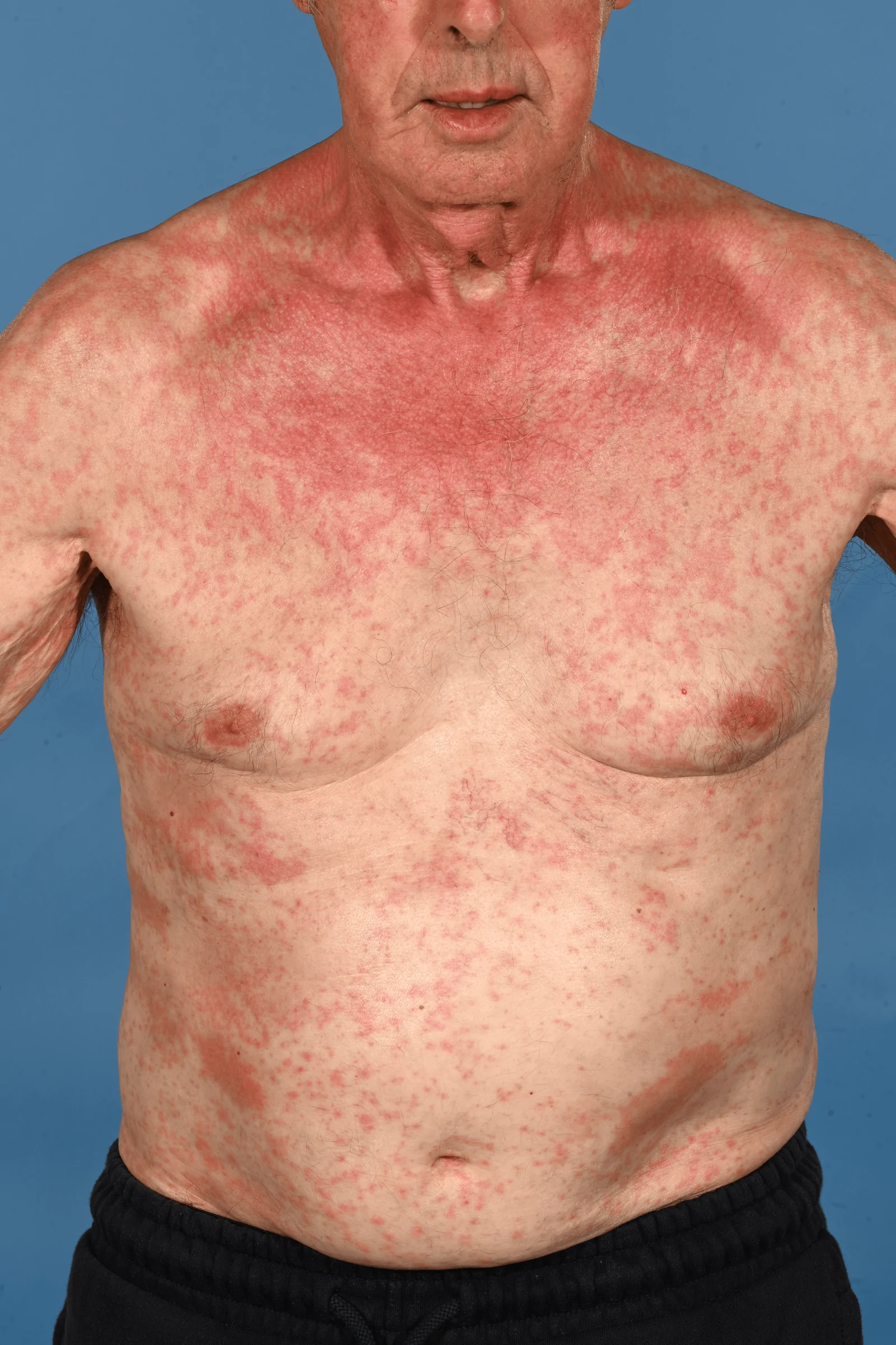
Clinical photograph showing an extensive rash

**Figure 5:**
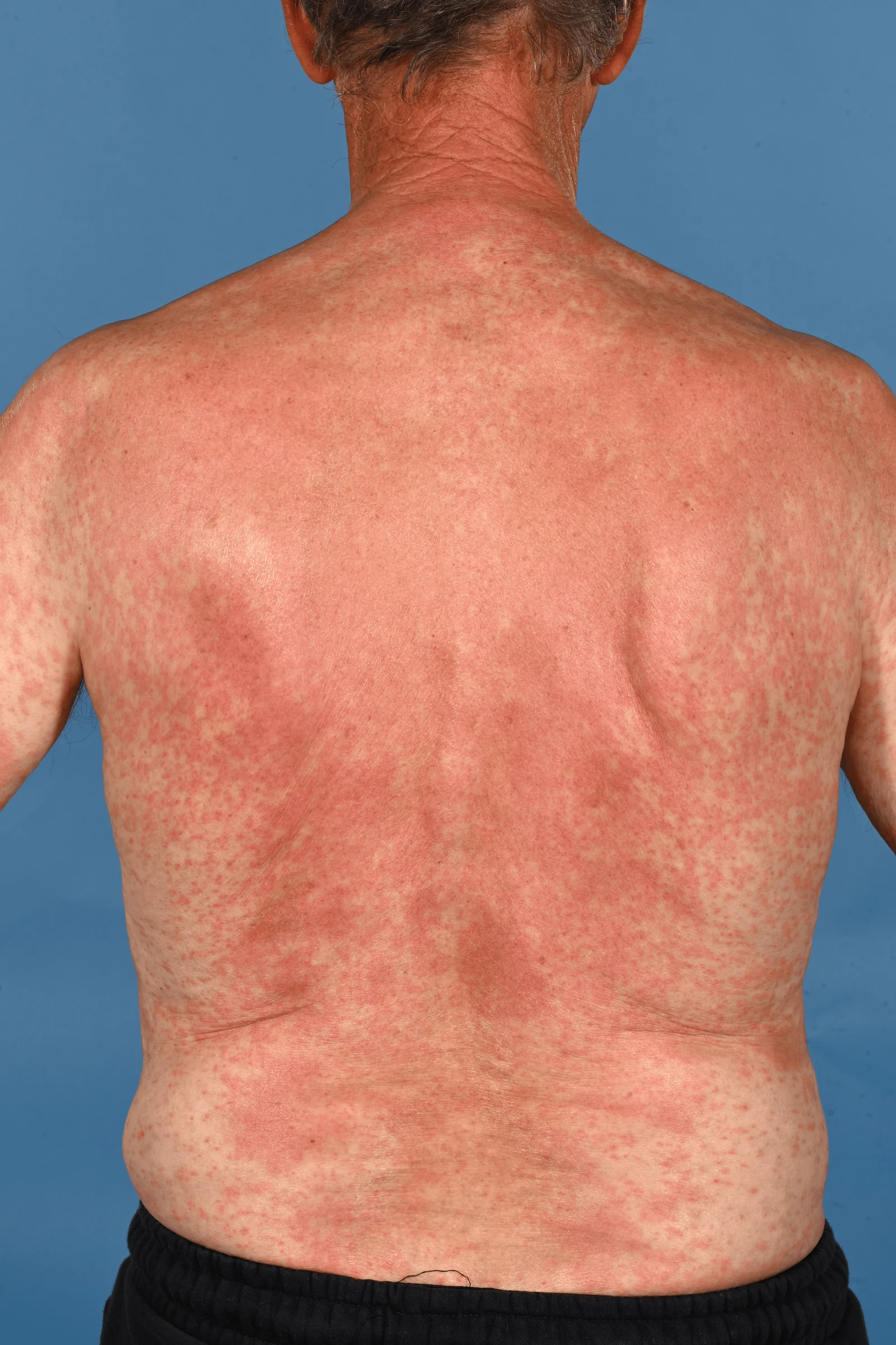
Extensive rash

Laboratory investigations revealed eosinophilia (absolute eosinophil count 1.10 × 10^9^/L), impaired renal function with an estimated glomerular filtration rate (eGFR) of 49 mL/min/1.73 m² (baseline >60), and an elevated creatinine level of 112 µmol/L. Liver function testing demonstrated marked transaminitis, with alanine aminotransferase (ALT) rising from 128 U/L to 343 U/L over one week (Table 1, 2). Serum calcium and electrolytes remained within normal limits. Urinalysis showed proteinuria, with a protein:creatinine ratio of 146 mg/mmol (reference range 0-15 mg/mmol), and ultrasound imaging of the liver was unremarkable.

**Table 1:** Laboratory investigations consistent with Bocquet's criteria eGFR, estimated glomerular filtration rate; ALT, alanine aminotransferase

Laboratory investigations	Result	Reference range
Eosinophil count	1.10×10^9^/L	0-0.4
eGFR	49 mL/min/1.73 m² (patient’s baseline >60)	>60
Creatinine	112 µmol/L	62-106
ALT	343 U/L (previous result - 128)	0-55

**Table 2:** Laboratory investigations after stopping apalutamide and following initiation of enzalutamide eGFR, estimated glomerular filtration rate; ALT, alanine aminotransferase

Laboratory investigations	One month after apalutamide discontinuation/on prednisolone discontinuation	One month after enzalutamide initiation
Eosinophil count	0.7	0.71
eGFR	55	50
Creatinine	114	125
ALT	62	32

A diagnosis of suspected DRESS syndrome was made. The patient was commenced on high-dose oral prednisolone (30 mg daily for seven days) with proton pump inhibitor prophylaxis. Steroid tapering was performed gradually over the subsequent four weeks, with the dose reduced every seven days to 20 mg daily, 10 mg daily, and 5 mg daily before discontinuation. On follow-up after apalutamide discontinuation, his rash had completely resolved at two weeks, liver function normalized within two months, and renal function returned to baseline by four months. His Eastern Cooperative Oncology Group (ECOG) performance status score remained excellent at 0.

Viral serology, including screening for herpesvirus, Epstein-Barr virus, and cytomegalovirus, was negative, thereby excluding viral reactivation as a contributing factor. Given the severity of his reaction, it was decided not to rechallenge with apalutamide. Instead, he was counseled and consented to treatment with enzalutamide as an alternative androgen receptor inhibitor.

Enzalutamide was well tolerated, without recurrence of the rash or biochemical deterioration. The patient continues on treatment at the time of writing.

## Discussion

DRESS syndrome is an uncommon but serious cutaneous adverse drug reaction. Diagnostic criteria vary, with three major scoring systems commonly used: the Registry of Severe Cutaneous Adverse Reactions (RegiSCAR), the Japanese Consensus Group criteria, and the Bocquet et al. criteria [7].

The RegiSCAR system requires more than 3 points for a DRESS diagnosis, based on hospitalization, suspected drug reaction, acute rash, fever, enlarged lymph nodes, involvement of one internal organ, or blood count abnormalities.

This patient did not meet the RegiSCAR criteria; however, he corresponded to a “possible” diagnosis of DRESS.

The Japanese Consensus Group criteria require the presence of all seven criteria, which include HHV-6 reactivation, prolonged clinical symptoms after discontinuation of the drug, a maculopapular rash developing three weeks after initiation of the drug, fever, lymphadenopathy, a rise in ALT, or blood abnormalities.

The presentation aligned more closely with Bocquet’s definition, which requires the triad of cutaneous eruption, hematological abnormality (eosinophilia >1.5 × 10^9^/L), and internal organ involvement, in this case, hepatic transaminitis exceeding twice the upper limit of normal [7].

Although apalutamide-associated rash is relatively common, DRESS syndrome has rarely been attributed to this drug. Current product information lists Stevens-Johnson syndrome (SJS) and lichenoid eruptions as adverse events of unknown frequency, with DRESS considered “not known” [8]. Nevertheless, scattered case reports have described apalutamide-induced severe cutaneous adverse reactions (SCARs), including one report of DRESS with a RegiSCAR score of 7, fulfilling the criteria for a “definite” diagnosis [9].

Given how well the patient remained during presentation and the overall clinical picture, the diagnosis best fit DRESS rather than other diagnoses. However, a more definitive diagnosis may have been established with the utilization of a dermatological biopsy. Apalutamide was considered the causative agent, as this was the only recent medication commenced. Prior to this, he had been well established on degarelix.

The pathogenesis of DRESS remains obscure. Proposed mechanisms include genetic susceptibility, particularly associations with specific human leukocyte antigen (HLA) alleles, altered drug metabolism, and viral reactivation, most notably human herpesvirus 6 (HHV-6) [10]. It is likely that a complex interplay of these factors contributes to disease expression.

Treatment of DRESS involves immediate withdrawal of the offending drug and initiation of systemic corticosteroids in cases with significant organ involvement. A slow tapering regimen is advised to prevent relapse. Additional immunosuppressive therapies may be considered in refractory or severe cases [7].

DRESS can have serious complications if not recognized and treated promptly. The mortality rate is 10%. One of the most common causes of death in DRESS is fulminant hepatic failure, but multiorgan failure can also occur. Myocarditis is another complication of DRESS. Other long-term complications include autoimmune disorders such as hemolytic anemia, thyroiditis, and systemic lupus erythematosus. These complications can develop even several years after DRESS and occur in approximately 16% of cases [7].

In this case, timely recognition of DRESS and initiation of systemic corticosteroids led to complete resolution of symptoms and biochemical recovery. Importantly, early discontinuation of apalutamide prevented further progression of organ dysfunction, highlighting the value of vigilance in oncology practice.

## Conclusions

Apalutamide is increasingly used in the management of advanced prostate cancer, with cutaneous adverse reactions being relatively common. However, DRESS syndrome remains an exceedingly rare complication, with only a handful of cases reported worldwide. This case contributes to the growing body of evidence suggesting that apalutamide may be a potential trigger for DRESS.

Given the potential severity of DRESS, pharmacovigilance and reporting of suspected cases remain crucial to improving recognition and guiding future management strategies.
